# Recent Advances in Polymer Nanocomposites: Unveiling the Frontier of Shape Memory and Self-Healing Properties—A Comprehensive Review

**DOI:** 10.3390/molecules29061267

**Published:** 2024-03-13

**Authors:** Huma Jamil, Muhammad Faizan, Muhammad Adeel, Teofil Jesionowski, Grzegorz Boczkaj, Aldona Balčiūnaitė

**Affiliations:** 1 Institute of Physical Chemistry, Polish Academy of Sciences, Kasprzaka 44/52, 01-224 Warsaw, Poland; humaj1137@gmail.com; 2 School of Chemistry, University of the Punjab, Lahore 54590, Pakistan; faizan.muhammad7777@gmail.com; 3 Institute of Chemistry, Technische Universität Chemnitz, Straße der Nationen 62, D-09111 Chemnitz, Germany; 4 Faculty of Applied Engineering, iPRACS, University of Antwerp, 2020 Antwerp, Belgium; muhammad.adeel@uantwerpen.be; 5 Institute of Chemical Technology and Engineering, Faculty of Chemical Technology, Poznan University of Technology, Berdychowo 4, 60-965 Poznan, Poland; teofil.jesionowski@put.poznan.pl; 6 Department of Sanitary Engineering, Faculty of Civil and Environmental Engineering, Gdańsk University of Technology, 11/12 Narutowicza Str., 80-233 Gdańsk, Poland; 7 EkoTech Center, Gdańsk University of Technology, G. Narutowicza St. 11/12, 80-233 Gdansk, Poland; 8 Department of Catalysis, Center for Physical Sciences and Technology, Sauletekio av. 3, LT-10257 Vilnius, Lithuania; aldona.balciunaite@ftmc.lt

**Keywords:** functional materials, materials design engineering, shape recovery, nanomaterials, nanocomposites, biomedical applications

## Abstract

Shape memory and self-healing polymer nanocomposites have attracted considerable attention due to their modifiable properties and promising applications. The incorporation of nanomaterials (polypyrrole, carboxyl methyl cellulose, carbon nanotubes, titania nanotubes, graphene, graphene oxide, mesoporous silica) into these polymers has significantly enhanced their performance, opening up new avenues for diverse applications. The self-healing capability in polymer nanocomposites depends on several factors, including heat, quadruple hydrogen bonding, π–π stacking, Diels–Alder reactions, and metal–ligand coordination, which collectively govern the interactions within the composite materials. Among possible interactions, only quadruple hydrogen bonding between composite constituents has been shown to be effective in facilitating self-healing at approximately room temperature. Conversely, thermo-responsive self-healing and shape memory polymer nanocomposites require elevated temperatures to initiate the healing and recovery processes. Thermo-responsive (TRSMPs), light-actuated, magnetically actuated, and Electrically actuated Shape Memory Polymer Nanocomposite are discussed. This paper provides a comprehensive overview of the different types of interactions involved in SMP and SHP nanocomposites and examines their behavior at both room temperature and elevated temperature conditions, along with their biomedical applications. Among many applications of SMPs, special attention has been given to biomedical (drug delivery, orthodontics, tissue engineering, orthopedics, endovascular surgery), aerospace (hinges, space deployable structures, morphing aircrafts), textile (breathable fabrics, reinforced fabrics, self-healing electromagnetic interference shielding fabrics), sensor, electrical (triboelectric nanogenerators, information energy storage devices), electronic, paint and self-healing coating, and construction material (polymer cement composites) applications.

## 1. Introduction

Polymeric materials are a widely discussed class of materials that have gained considerable attention in the last century owing to their vast importance. Advancements in their properties has increased to an extent that they exhibit intelligent healing phenomena similar to those observed in natural plants and animals, earning them the name “smart polymers” or “stimuli-responsive polymers”. A shape-changing polymer is an example of a stimuli-responsive polymer, named for its ability to change shape at the molecular or microscopically visible level [1]. Another class of polymer, known as shape memory polymers (SMPs), have the ability to retain their shape after the application of a temporarily applied force. The main difference between these two classes of polymer is that the shape-changing polymers exhibit a non-modifying phenomenon after their fabrication, whereas the modifying ability of SMPs can be altered [2]. Due to the application of external force, SMPs exhibit a shift in shape from permanent to temporary, and this behavior is referred to as the dual-shape memory polymer effect [3].

The strain on SMPs works in three steps. First, the strain is applied above the glass transition temperature, followed by the deformation of the polymer to its desired shape near room temperature in the second stage. The final stage involves the removal of the applied force and return of the polymer to its original shape above the glass transition temperature [4]. Owing to the ability of shape memory polymers to withstand and retain vast amounts of pressure, they are suitable for use in strict environmental conditions. They have versatile applications in various fields, such as space structures, medical applications, due to their lightweight nature, and textiles for breathable clothing [5]. The active controllable temperature of SMPs can be maintained by changing their composition and the degree of crosslinking. Additionally, the curing of SMPs can be achieved at low temperatures, resulting in advantageous properties. It is also possible to produce a microelectromechanical system using SMPs by coupling techniques such as casting, extrusion, and blow molding with photolithography and micro-casting [5,6,7]. 

To enhance the recoverable strain applied to the SMPs, reinforcements are added for structural applications. Some studies have demonstrated that the addition of nano-structures to polymers can significantly increase their interface (stiffness) properties, whereas micro-structures typically increase their strength and modulus [8,9]. SMP nanocomposites possess a significant importance in the fields of polymers since they are considered as a novel class of composites with at least one dimension in the nano-scale range, which increases their surface area. This is crucial for their interaction between nanoparticle fillers and the polymer matrix, including the exclusion of defects caused by micro-scale fillers [10]. The potential benefits of using nanofillers such as nanoparticles, nanotubes, nanowires, nanofibers, and nano-rods in SMP nanocomposites are becoming increasingly important. In several studies, resins are utilized in the fabrication of SMP nanocomposites through vacuum-assisted resin transfer molding (VARTM), resin transfer molding (RTM), filament winding, and resin film infusion (RFI) techniques [11,12,13]. To improve the properties of SMPs, nano-sized SiO_2_ is added to the polylactide-based polyester (PLAE), resulting in increased mechanical strength, shape flexibility, and recovery temperature, particularly in relation to body temperature. The low trigger temperature of the composite makes it particularly applicable in the biomedical field [14]. Another study demonstrated that the addition of carbon nanofibers to the epoxy shape memory nanocomposite resulted in a 1.0 wt% increase in Young’s modulus and an improvement in the product strength [15]. The area of research surrounding 3D printing technology has gained considerable attention due to its vast freedom of design, rapid engineering, and high resolution. When applied to polymer composites, the 3D printing technique can greatly enhance the properties of SMPs. For example, digital memory polymers exhibit distinct responses to various stimuli [16].

**Figure 1:**
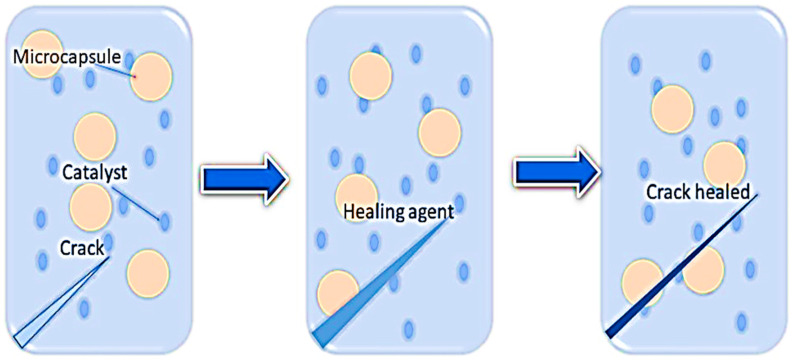
Autonomous self-healing mediated with embedded microcapsules.

Apart from shape memory polymer nanocomposites, another class of intelligent polymer nanocomposites includes self-healing polymer (SHP) nanocomposites, which are being widely recognized in the scientific community owing to their broad range of applications. Synthetic SHPs have the ability to recover their properties autonomously and spontaneously after damage, fracture, or corrosion. Numerous studies have reported various types of healing materials, including elastomers, thermosets, supramolecular polymers, thermosetting polymers, nanocomposite polymer coatings, and polymer composites. SHPs are classified into two categories: autonomous and non-autonomous SHPs [17,18,19,20] (Figure 1). Autonomous self-healing composites possess an increased healing potential owing to the addition of nanostructures, which localize in nanoscale cracks and repair material in patches. Studies have reported that the healing properties of nanocomposite can reach up to 75–100% compared to the original nanocomposite materials [17,21]. By the addition of different nanomaterials into the SHPs and SMPs, the magnetic, electrical, and mechanical properties of these polymer nanocomposites change as per the stimulus. In the biomedical field, these polymers depict distinct applications, such as tissue engineering, cardiac valve repairing, bone tissue, wound healing, anti-bacterial properties, and so on [22,23]. This review will cover the properties of SMPs and SHPs, their healing mechanism, and their biomedical applications.

## 2. Self-Healing Polymer Nanocomposite (SHPs) 

**Figure 2:**
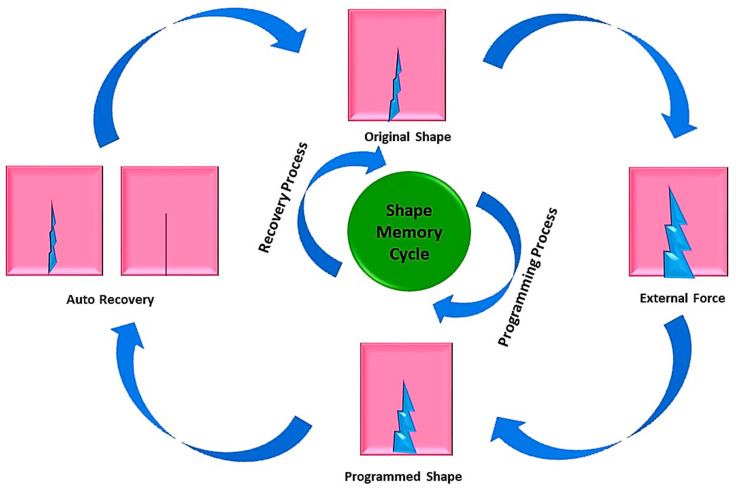
Self-healing process by internal forces.

Functional polymers can benefit significantly from the addition of fillers and reinforcements, such as clay, cellulose, metal, carbonaceous materials, and silica, for various applications. These fillers can substantially improve their properties, including the toughness, Young’s modulus, and strength of the polymeric matrix. The healing ability of the polymer nanocomposites can be activated via various methods, including the Joules effect. The Joules effect activates the thermal healing ability of the polymeric materials as an electrical current passes through the nanostructured network [24]. Self-healing materials are characterized by their intrinsic and extrinsic properties, which have been extensively reported. The intrinsic system involves block copolymers containing reactive functional groups that exhibit reversible bond interactions covalently or non-covalently upon applying external stimuli [25,26]. On the other hand, extrinsic properties demonstrate a limited amount of healing capacity when stimuli surpass a threshold [25,27]. Self-healing can occur automatically through the interference of external stimuli, which is advantageous in various applications, including coatings, energy, electronics, and biomedical devices [28,29,30,31]. The self-healing process in the polymer matrix arises from the cumulative effect of chemical interaction [32], physical association [33], crosslinking [34], chain movement [35], and polymerization processes [36] (Figure 2). Subsequently, several factors influence the healing process of polymer matrices, including chain entanglement, chemical repair, and polymerization processes [30,31,37,38,39]. 

The incorporation of nanostructures into a polymer matrix enhances the polymeric properties, which can expand novel approaches for the development of high-performance multipurpose materials. However, achieving a homogenous dispersion of nanomaterials is necessary for the production of high-performance, multipurpose, self-healing nanocomposites [40]. Moreover, factors including morphology, nanoparticle content, and microphase separation can enhance the self-healing properties of polymers [41].

### Effect of Nanocomposite Fillers on Healing Properties

The addition of nanomaterials to polymeric materials helps to improve the healing ability of the polymeric materials.

Wu et al. added polypyrrole nanoparticles to produce thermoplastic polyurethane (TPU) SHPs. The thermal stability of the resulting polymer was increased with the addition of polypyrole (PPy) augments, attributable to the hydrogen bonding with ester and urethane bonds, with nearly 100% healing ability. This high healing ability is attributed to the fact that TPU/PPy can be heated under NIR [42]. Carboxyl methyl cellulose (CMC) nanocomposites based on carbon nanotubes (CNTs) containing murexide (M) salt exhibited a self-healing ability at ambient temperature and 80% humidity, with the cut section merely needing to be touched for healing to occur within 48 h. As the amount of CNT in the polymer increased, the Young’s modulus was enhanced and elasticity was reduced. DMA cycles showed a 70% healing ability at 20–30 °C, with an increase in temperature by 60–100 °C resulting in a 50% reduction in healing values. Thermal gravimetric analysis of the sample revealed that the addition of M salt increased the thermal stability of the polymer in the range of 400–580 °C and enhanced the thermal resistance [43]. Ubaid et al. demonstrated that the incorporation of titania nanotubes into epoxy monomer (EM) and dodecylamine (DDA) enhanced the self-healing and autonomous corrosion resistance of the epoxy and polymer. Titania nanotubes accelerated the healing ability to 5 days, with a healing efficiency of 63%. This healing phenomenon occurred through the release of EM from the broken nanotubes, which then made contact with the amine group on the nanotube, functioning as a curing agent for subsequent crosslinking. Additionally, the presence of DDA in the polymer matrix enhanced the anti-corrosion properties. The reinforcement provided by the titania nanotubes also had a significant impact on the mechanical properties of the material [44]. The addition of a novel graphene-epoxy nanocomposite as a macrolinker using *β*-cyclodextrin/graphene (CD-G) incorporated into the epoxy linkage also improved the mechanical properties. The incorporation of 1% CD-G resin produced improved healing properties, which repaired defects in the polymer film, including enhanced corrosion resistance and anti-penetration properties [45]. Similarly, polyurethane graphene oxide nanocomposites also demonstrated 70% healing ability in 48 h in stress–strain analysis, along with anti-corrosion properties [46]. Self-healing epoxy coatings also depict a distinct anti-corrosion ability when loaded with mesoporous silica (MSP) and polyethyleneimine (PEI). MSP and PEI worked as inhibitors and helped in blocking the defects present in the epoxy coating, hence enhancing its anti-corrosion ability with healing ability [47].

## 3. Methods of Self-Healing in Self-Healable Polymer Nanocomposites

Several interactions are responsible for the healing process of SHP nanocomposites (Table 1). These aspects are discussed in the following paragraphs.

### 3.1. Diels–Alder Reaction and Thermo-Responsive

The Diels–Alder reaction is a process where a diene and a dienophile react to produce cyclohexene. The reaction can be reversed through a retro-Dies–Alder mechanism, which causes the cyclohexene to break down into the initial reactants. Both reactions are temperature dependent, and an increase in temperature favors the Diels–Alder (DA) reaction while a decrease in temperature results in a retro-Diels–Alder (rDA) reaction [48]. This temperature sensitivity impacts the structure of the polymer nanocomposites, augmenting their self-healing efficiency and mechanical properties. However, to facilitate DA and rDA reactions, certain nanofillers can be used in the polymer matrix.

#### 3.1.1. Multi-Walled Carbon Nanotubes (MWCNT)

**Figure 3:**
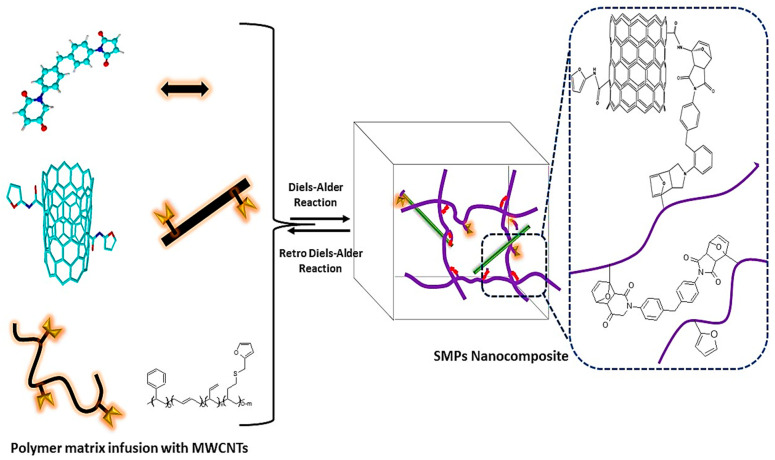
Dies-Alder reaction in SHPs.

In the healing mechanism following Dies-Alder reactions, MWCNTs act as both diene and diebophile, which facilitates the DA reaction. The incorporation of furfuryl-terminated MWCNT (MWCNT-FA) into furfuryl-joined styrene-butadiene rubber improves the self-healing and mechanical properties (Figure 3). MWCNT-FA plays a dual role as a healing agent and reinforcement of the material. MWCNT facilitates DA reactions with maleimide and furan moieties. The synthesized polymer nanocomposites showed thermal responsiveness, with maleimide undergoing the DA reaction as the temperature cooled from 160 °C to 74 °C and the rDA reaction as the temperature was further lowered from 80 °C. Furthermore, the addition of magnetic nanoparticles into the polymer nanocomposites imparted magnetic behavior, which acted as a healing stimulus in the presence of a magnetic field [49]. Utilizing a magnetic copolymer for the healing mechanism results in supramagnetic behavior when subjected to an applied magnetic field, which diminishes upon the removal of the magnetic field. Polycaprolactone-poly(furfuryl glycidyl ether) and iron oxide nanoparticle-decorated carbon nanotubes exhibited self-healing properties via the rDA mechanism. The DA reaction occurred between the diene of MWCNT and the dienophile of the furan group of polycaprolactone-poly(furfuryl glycidyl ether) (PCLF), forming PCLF/IONPs-MWCNTs DA adducts. The adduct depicts supramagnetic behavior. The hybrid polymer system exhibits thermal reversibility in the DA system, and at 140 °C liquidity is observed, attributable to the rDA reaction disassembling the DA linkage, thereby increasing the healing ability [50]. Multi-walled carbon nanotube (MWCNT) polymer (furan-derived PK30 and the aromatic bismaleimide PK30xFUyA2Pz/B-Ma/MWCNT) thermosets exhibit self-healing mechanisms by means of a three-dimensional network via the DA reaction. The system showed the ability for both DA/rDA and hydrogen bond-induced healing. The DA reaction reduced the crosslinking density of the polymer. Hot compression molding of the sample favored the rDA mechanism, indicating that the material is reversible and recyclable at temperatures above 120 °C. The healing ability of the polymer was increased by applying pressure to increase the contact between the layers. The best thermo-healing of the sample occurred at 150 °C for 24 h after annealing and molding [51]. In recent studies, it was reported that CNTs can be directly employed in the polymer matrix without prior modifications, which is a promising approach. Guo et al. reported that CNTs can be directly integrated with furan-pendant polymers, which portrayed active temperature-reliant behavior. The side-chain reaction of the polymer increased the irreversible increase in the modulus, with appreciable reversibility below 120 °C. The polymer nanocomposite showed healing ability towards both electrical and thermal stimulus, with 5% loading of CNTs demonstrating 90% efficacy. The defect sites in carbon play a crucial role in the DA reaction because defect sites result in more active sites, enabling larger cross-linked mesh in the polymer matrix [52].

#### 3.1.2. Graphene Derivatives

In addition to CNTs, functionalized graphene oxide has also been shown to be a promising filler in SHP nanocomposites, with a healing ability greatly dependent on the DA/rDA mechanism. In a study by Lin et al., terminal maleimide groups were synthesized for crosslinking, and furan groups were attached to polyurethane (PU) as pendant groups to prepare self-healing nanocomposites. Silver nanowires (AgNWs) were deposited on the surface of the matrix material, functioning as a conductive material, while graphene oxide material (rmGO) was added as a filler, imparting healing properties to the matrices following the DA reaction mechanism. The cumulative effect of both AgNW and rmGO imparted the polymer with an exceptional NIT-induced healing ability. Moreover, maleimide reacted with the furan group, forming adducts, which promoted compatibility between the polymer and the rmGO and demonstrated a healing ability (electrical and structural) stimulated by NIR radiations. The composites comprised of rmGO showed a fast upward trajectory regarding temperature, exhibiting the excellent photo-thermal effect of rmGO, leading to high absorbance at 808 nm NIR radiation and high healing impact [53]. The size of the GO also impacts the mechanical and self-healing properties. Smaller GO particles significantly improved the self-healing and mechanical properties of PU by increasing the crosslinking ability, while larger GO particles interfered with the DA bond. However, increasing the amount of functionalized graphene oxide (mGO) reduced the efficiency of the mechanical properties, as the maleimide group decreased the flexibility of the polymer chain [54]. In multichannel self-healing material (FGE-EDR/MDPB@FGO) comprised of mGO, healing abilities can reach 106% in 5 s, 133% in 60 s, and 90% in 60 s following IR, heat, and microwave stimulus, respectively. This high healing ability is attributed to the mGO, which can transfer the heat rapidly to the damaged area, facilitating the DA polymerization process. DA polymerization increased the heat acceptance of the polymer matrices. The study also revealed that under IR and microwave radiation, DA and rDA occur via the conversion of radiation into thermal energy, followed by the cleavage and repair of the bond via the DA mechanism [55]. Nanocomposite polymers composed of furfurylamine (FA)-modified diglycidyl ether of bisphenol A (DGEBA) and furfuryl-functionalized aniline trimer-modified graphene (TFAT-G) with bismaleimide (BMI) showed that only a small proportion of TFAT-G (1%wt) imparts the high tensile strength of 233%. The polymer also showed a high electrical conductivity of 89%, along with reusability. However, the properties of the polymer deteriorate if the graphene is poorly dispersed, which causes a lack of interfacial interaction [56]. The adduct of BMI and graphitic nanoplatelets (GNPs) showed an enhanced flexural strength of almost 21%, and healing efficiency reached almost 75% [57].

#### 3.1.3. Other Nanofiller

Self-healing polyurethane/halloysite nanocomposites with halloysite nanotubes exhibited improved mechanical properties and healing efficiency. The polymer nanocomposite showed a high healing efficiency of 90%. The addition of 1%wt HNTs resulted in a significant increase of up to three times in Young’s modulus and tensile strength at room temperature. However, the elongation at break decreased due to the increase in hydrogen bonding content, which also makes them a candidate for self-healing coatings. Furthermore, linking graphene with magnetic nanoparticles induced a shielding ability from electrical and magnetic fields in the resulting materials, including a near complete healing ability in a short period of time [58]. Nevertheless, not all nanofillers can improve the mechanical properties of the polymer nanocomposite, and some fillers may even have negative impacts. This negative impact is because of molecule stereochemistry, which is an influential factor in forming the 6-membered ring, which is the main attribute of the DA reaction. Adding silane nanoparticles as reinforcements in either 2-furyl-(undecenyl)-11-triethoxysilane or 3-maleimidopropyltriethoxysilane as coupling agents provides branched and linear chain polysiloxanes. The comparison of both polymeric materials showed that a polymer with terminal branched polysiloxanes was preferred due to the larger space around the DA entities. However, the polymer did not show considerable enhancement in healing ability due to poor chain mobility and steric hindrance, which barred the polymer from undergoing the 6-membered cycloaddition reaction. However, an alcoholic environment was found to increase the self-healing ability of the polymer. The DA reaction also increased the viscosity of the polymer material due to the crosslinking of nanoparticles with polysiloxane [59]. Polymers based on poly(ε-caprolactone)/clay nanocomposites, maleimide, and end groups comprised of poly(ε-caprolactone) (PCL) and furan-functionalized montmorillonite (MMT) also show a self-healing ability. The addition of MMT acted as a nanofiller in the polymer, resulting in changes to its thermal and mechanical properties. The polymer exhibited an increase in thermal stability compared to ungrafted PCL [60]. Organically modified nanoclay used with carbon black (Cloisite 15A) is also employed as a nanofiller for self-healing polymer nanocomposite material. Cloisite 15A coupled with poly(propylene oxide) (PPO) works synergistically, improving the electrical conductivity and healing ability of the polymer nanocomposite. The synergistic effect of the Cloisite 15A enhanced the healing efficiency to 88%, while the electrical conductivity of the polymer increased by 70%. It was noted that by increasing the compatibility between the polymer and Cloisite (30A), the mechanical properties exhibited an upward trajectory by increasing the extent of crosslinking, providing a healing efficiency of 193%, but at this concentration the electrical conductivity of the polymer nanocomposite reduced significantly (28%) [61].

### 3.2. Quadruple Hydrogen Bonding (QHB)

**Figure 4:**
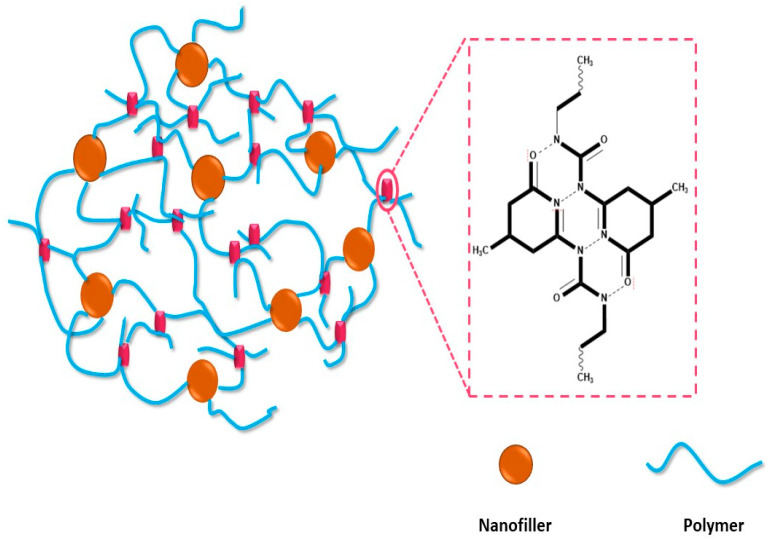
QHB between polymer chain and nanofiller.

Hydrogen bonding (HB) has the ability to enhance the mechanical properties and self-healing capability of self-healing materials under ambient conditions. Owing to this property, polymer materials can be engineered to certain desired properties, such as increased toughness and reliability [62]. Materials rich in HB are able to recover their electrostatic interactions gradually, which results in a high healing ability. Various types of polymers, including thermos-responsive polymers, crystalline supramolecules, light healable supramolecules, electrodes based on printed and flexible carbon nanotubes, and supramolecular architecture, rely on HB for enhancing their properties. The majority of the molecules that exhibit HB healing abilities contain ureidopyrimidinone (UPy), which can be introduced into the polymer by reacting 2-amino-4-hydroxy-6-methylpyrimidine (AHMP) with isocyanate (Figure 4) [63]. 

Lin et al. reported the synthesis of a supramolecular polymer nanocomposite through the combination of SiO_2_ functionalized with ureido-pyrimidone (UPy) and polycarbonatediol end-capped by UPy. The SiO_2_-UPy interacted with the UPy via quadrupole HB, which enhanced the Young’s modulus and tensile strength by 198% and 292%, respectively. A small proportion of UPy was enough to produce a significant self-healing ability in the polymer nanocomposite. QHB helps in assembling the UPy groups, forming a high-molecular-weight polymer nanocomposite that yields a higher degree of tensile strain and tensile stress. HB also positively impacts the Young’s modulus and tensile strength. Increasing the amount of SiO_2_-UPy leads to aggregation, which harms the mechanical properties of the polymer nanocomposite [64]. PU decorated with UPy exhibits self-healing properties at room temperature. The T_g_ of the polymer nanocomposite showed a tunable property with constituent yield. UPy impacted the healing efficacy of the nanocomposite depending on the added amount. A higher amount of UPy results in zilch intermolecular crosslinking due to capped end groups. This results in the facilitated movement of the polymer chains at room temperature, allowing stronger QHB, resulting in a high healing ability. However, an excessive amount of UPy restricts the chain movement, hence lowering the healing ability. The incorporation of UPy motif resulted in an increase in the Young’s modulus of up to 1.58 ± 0.30 MPa, while there was a decrease in elongation at break of 4 ± 0.1% from 1286 ± 42. However, with a moderate amount of UPy and PPG-triols content, the Young’s modulus increased to 3.02 ± 0.10 MPa and elongation at break increased to 1508 ± 85%. The self-healing property was considerably enhanced, with a healing efficiency of 97% within 1440 min [63]. The addition of magnetic Fe_3_O_4_ nanoparticles to a prepared polymer nanocomposite comprised of poly(*n*-butyl acrylate) imparts superparamagnetic properties and a higher saturation moment to the polymer nanocomposite. However, QHB also reduces the free motion of the polymer chains. Furthermore, the addition of 2-ureido-4[1H]-pyrimidinone methyl methacrylate (UPyMA) during copolymerization facilitated hydrogen bonding interactions within the polymer matrix, which enhanced the thermomechanical properties of the polymer matrix. For instance, the addition of 9.7 mol% UPyMA increased the glass transition temperature to −2 °C and elongation at break to 275%, indicating the ductility of the nanocomposite molecules owing to the addition of P(MA-co-UPyMA)@Fe_3_O_4_ NPs. Furthermore, a high UPyMA content enhanced the self-healing property at 40 °C [65]. In polyhedral oligomeric silsesquioxanes (POSS-POSS), QHB enhances the T_g_ from −52 °C to −48 °C, with elongation at break increased by 450% in 15 hrs at 40 °C [66]. Waterborne polyurethanes (WPU-CHZ-NAGA) containing irregular 6-fold and diamide hydrogen bonds showed much superior mechanical properties at room temperature. The polymer nanocomposite exhibited a tensile strength of 36.58 MPa, toughness of 125.82 MJ·m^−3^, tearing energy of 81.2 kJ·m^−2^, and healing ability of 92%. These high mechanical properties occur through the synergistic effect of ethanol and hierarchical HB due to the presence of 6-folds of HB. This poses a high degree of HB, which also increases the adhesive ability of the polymer nanocomposite, along with the strengthening and toughening effect [67]. QHB can also be introduced into the material, such as polyaniline induced with polyacrylamide (PAA) and oxidized nanocellulose. Equal amounts of polymer and nanocomposite provide the matrix with high mechanical properties, such as a healing ability of more than 90%, electrical conductivity of up to 47.22 μS/cm, and a toughness of 1.62 MPa. The polymer also demonstrates considerable reproducibility after many cycles, with the recovery of mechanical properties [68].

### 3.3. π–π Stacking Interaction

Numerous studies have reported that increasing the number of π–π interactions has been found to improve the mechanical properties of polymeric materials. However, achieving a 100% healing effect in such interactions typically requires a longer time and higher temperatures [69,70].

Controlling the nanoparticle constituency promotes better π–π stacking interaction, resulting in improved healing efficacy and mechanical properties, without disturbing the tensile modulus in some cases, as reported by Vaiyapuri et al. They synthesized a SHP nanocomposite comprising of a mix of polyamide functionalized by pyrene and gold nanoparticles functionalized with pyrene and polyamide (P-AuNPs), which had π–π stacked interactions due to the electron-filled pyrenyl and electron-deficient polyimide. The addition of 15 wt% P-AuNPs in the polymer matrix greatly improved the properties of the resin, but a controlled amount of nanoparticle loading produced more acceptable results. A film with 1.25 wt% P-AuNPs showed healing properties at 50 °C in 10 min, with a healing efficiency of 108% and an observable increase in the modulus of the film after healing, attributed to a mechanically stronger network of supramolecules. The controlled sample had a notably similar tensile strength modulus to that of the supramolecule mix without P-AuNPs, indicating that the π–π stacking interaction between the polymer and P-AuNPs was significantly responsible for the enhanced mechanical and healing properties of the polymer nanocomposite matrix [71]. Grating rGO with polymer comprised of polystyrene (PS), poly(styrene-co-4-cyanostyrene) (PSCN), and poly(styrene-co-2-vinylnaphthalene) (PSNP) via π–π stacking increases the T_g_. Nanocomposites composed of 4.0 wt% RGO/PS, RGO/PSNP, and RGO/PSCN exhibited an increase in T_g_ of 14.3 °C, 4.4 °C, and 25.2 °C, respectively. The π–π interaction was formed only by the aromatic ring, but substitution leads to changes in the electronic cloud density on the phenyl ring, leading to an increase in T_g_. A stronger π–π interaction leads to greater T_g_ [72]. π–π interaction helps in shrinking the healing ability of the polymer matrix. Hyperbranched PU with 3-aminopropyltriethoxysilane-modified graphene oxide sheets (Si-GO) provides a significant enhancement in mechanical properties with only 2 wt% of nanofiller into the polymer matrix. With a small amount, elongation at breakage was increased to ~206%, tensile strength to ~247%, and toughness to ~330%. Furthermore, the polymer exhibited almost 100% healing efficiency within 50–60 s under sunlight. The elasticity of the polymer increased by increasing the nanocomposite content due to the lengthening of the π–π interaction between the polymer-coiled chain and the Si-GO, and only a small proportion of the nanocomposite is enough to enhance the interaction [73]. In the pursuit of making new SHP nanocomposites, polyurea is always a challenge because introducing new functional groups jeopardizes the mechanical properties of the polymer. However, preparing polyurea polymer using pyrene and amino groups increases the tensile strength of the polyurea to 41.4 MPa (530%). The high tensile strength is caused by the π–π stacking effect. Additionally, using pyrene in the polymer also imparts the polymer with UV shielding and a fluorescent effect. Polyurea made of pyrene presents high polarity, which helps in the dispersion by nanocomposite (graphene) π–π interaction. Graphene with polyurea increases the dielectric constant by three and presents a healing efficacy of 89.0%. The polymer nanocomposite showed an elongation at breakage of 66.3%, with a 90% strain recovering ability [74]. A vitrimer epoxy-based polymer with mGO and 4-aminophenyl disulfide (4-AFD) presents a healing efficiency of 110% and a high T_g_ range due to enhanced interfacial linking. Increasing the mGO in the polymer matrix increases the mechanical properties along with T_g_, including thermal stability and cyclability [75]. Using a polymer with biocompatible polyacrylamide (PAM) and polydopamine-modified carbon nanotubes (PDA@CNTs) provide a healing efficacy of 97.3%, along with a 400% strain retention ability. The polymer exhibited firm adhesion, biocompatibility, and sense-gauge ability due to HB and π–π interaction [76].

### 3.4. Metal–Ligand Interaction

**Figure 5:**
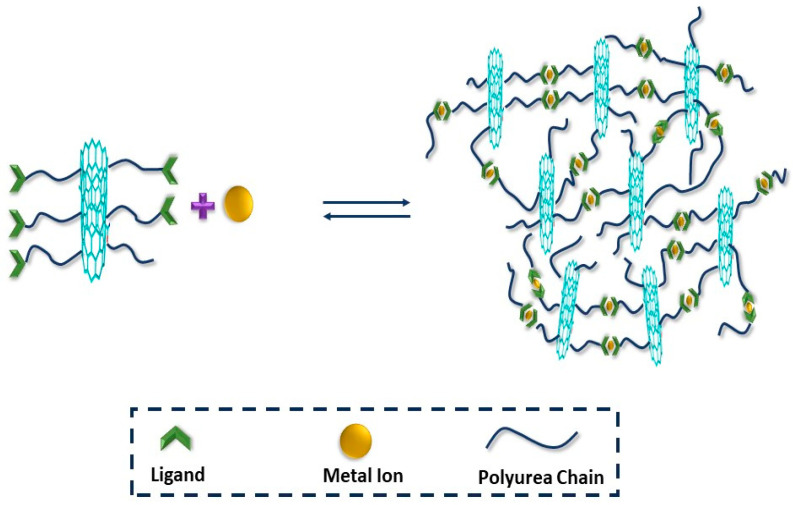
Copolymer nanocomposite crosslinked with metal, forming a metallo-supramolecular polymer nanocomposite.

Metal–ligand coordination polymer nanocomposites consist of a metal ion at the center, surrounded by polymer monomers (Figure 5). These materials possess optical and stimuli-sensitive properties due to the presence of the metal. The bonds between the metal and the polymer matrix are dynamic non-covalent bonds, resulting in the stiffness and strength of the polymer nanocomposite [77]. In metal–ligand interaction, moderate bond energy causes association dissociation between metal and ligand, and this also induces a healing ability to the polymer nanocomposite. A PU ligand terminated with terpyridine polymerized on the surface of MWCNT dynamically cross linked by zinc metal ions (Zn^2+^) displays strength, toughness, and elasticity. The healing process follows the complexation–decomplexation mechanism, whereby ionic charges agglomerate into clusters through various complex interactions with negatively charged ions. The polymer nanocomposite showed an increase in tensile strength from 14.2 MPa to 22.8 MPa, and an elongation at breakage from 620% to 1076%. The composite exhibited a healing efficiency greater than 93% towards various stimuli, including NIR (4.2 mW·mm^−2^, 30 min), and at a comparatively low temperature of 90 °C within an hour. Healing of the polymer occurs above 80 °C, which is due to the metal–ligand decomplexation reaction that occurs above this temperature [78]. The polymer (methylimidazole/methylthioether-containing squalene (SQ) nanoparticles, (MI/MT-SiO_1.5_)) displayed a good Young’s modulus of 2.13 GPa and 80% healing ability at 50 °C within 24 h. After healing, the polymer retained the same mechanical properties as the pristine materials. The polymer required milder healing (lower temperature) due to the irreversible behavior of the zinc–imidazole interactions and the amorphous nature of SQ nanoparticles. The self-healing ability of the polymer nanocomposite was significantly improved upon exposure to infrared radiation. A higher crosslinking density provides a high modulus of 2.13 GPa and a tunable toughness [79]. The addition of only 1.1% CNT into supramolecular CNTsx-g-CPy/Zn exhibited dynamic properties such as a maximum stress of 1.68 MPa and an elongation at breakage of 450%. The photothermal alteration of carbon nanotubes effectively activated the dissociation and association mechanism between the cross-linked bonds of VI and Zn^2+^, leading to a dynamic self-healing ability (Figure 5). The polymer exhibited a special crosslinking behavior that induced a high degree of flexibility of 1250%. The metal–ligand polymer nanocomposite demonstrated an increase in T_g_ and mechanical properties due to the polymer chain’s operation at higher temperatures. The polymer showed remote self-healing properties when activated by NIR, with a recovery of 93.6% in only 6 s [80]. Similarly, the addition of magnetic nanomaterials to polymer nanocomposites enhanced the remote healing ability, mechanical capabilities, and magnetic abilities. The Fe_3_O_4_x-g-CPy/Zn polymer matrix exhibited excellent magnetic, IR, and thermal-induced self-healing properties. The matrix showed a maximum stress of 1.66 MPa and a strain breakage of 300%. The stress at breakage of the polymer nanocomposite was enhanced by increasing the Fe_3_O_4_ contents due to the improved compatibility between the polymer matrix and the nanoparticles. The polymer depicted exceptional remote self-healing properties due to magnetocaloric and photothermal transformation, which stimulated a reversible and dynamic metal–ligand crosslinking mechanism between Zn^2+^ and the imidazole complex. Fe_3_O_4_ NPs converted NIR light into heat, facilitating the self-healing properties. Although the polymer’s healing efficiency was not remarkable, adjusting the Fe_3_O_4_ content and healing time increased the self-healing efficiency. After prolonged heating, the polymer achieved a 96.2% healing efficacy, while under NIR laser this efficacy time decreased to only 80s, with a healing efficiency of 89.2% [81]. The activity of some catalysts can bring the healing ability of the polymer nanocomposite to room temperature. In a recent study, Sanka et al. reported the formation of an epoxy-based SHP nanocomposite with a dispersed N-doped graphene-immobilized copper catalyst. The catalyst showed higher interaction with the polymer nanocomposite and brought the healing temperature down. The catalyst depicted a higher catalytic ability by the prevention of agglomeration because Cu^2+^ forms a coordination bond with N-doped-rGO, which causes a high electron transfer ability. The polymer after catalyst incorporation exhibited 100% healing at 60 °C in 36 hrs, while 96% healing occurred at room temperature. The polymer also showed an electrical conductivity of 5 × 10^−2^ S·cm^−1^, which can be attributed to nitrogen presence [82].

**Table 1:** Different self-healing mechanisms of polymer nanocomposites.

Self-Healing Reaction	Nanomaterials Used	Polymer	Glass Transition Temperature T_g_	Self-Healing %Age	Self-Healing Time and Temperature	Characterization Methods	References
Diels–Alder/Retro-Diels–Alder Reaction and Thermo-Responsive	Silane	2-furyl-(undecenyl)-11-triethoxysilane and 3-maleimidopropyltriethoxysilane	Nil	Nil	12 h, 60 °C	DSC, TGA, NMR, FTIR, DLS, TEM, BET	[59]
	HNTs	Natural Rubber	−65.17 °C	98.4%	Room Temperature, 10 mins	FESEM, SEM, HRTEM, XRD, DLS, FTIR, DSC	[83]
	GNT	PU, FD	50 °C	Nil	1 h, 110 °C	FTIR, DSC, TGA, FESEM, HNMR, Raman Spectroscopy	[84]
	GO	FGE-EDR/MDPB@FGO)	80–140 °C	90%-H-H 106%-IR-H 133%- MW-H	110 °C 60 s 5 s 60 s	Raman, XRD, TGA, XPS, SEM, TEM, EDS, HRTEM, SAED, DSC, FTIR	[55]
	rmGO	PU-EDM/rmGO	80–140 °C	Enhanced from 62 %	48 h, 65 °C	XRD, FTIR, DSC, SEM, UV-VIS, ASTM	[53]
	IONPs-MWCNTs	PCLF/BMI/IONPs-MWCNTs	Nil	Nil	140 °C	ATR-FTIR, XRD, HNMR, TGA, VSM, TEM, DSC	[50]
	GO, mGO	PU	Nil	98%	25 °C	FTIR, SEM, TGA, XPS	[54]
	B-GNPs	ER	Nil	87%	130 °C, 2 h 80 °C, 2 h	FESEM. ASTM, FTIR, TEM, HNMR, DSC, TGA, Raman Spectroscopy, EDX	[85]
	HNTs	PU	50 °C	90%	90 °C, 5 min 65 °C, 48 h	TGA, FTIR, SEM, XRD, DSC, MSSM.	[86]
	rGO/Fe_3_O_4_	mFPU	Nil	99%	900 W 10 min	FTIR, SEM, EDAX, Raman spectroscopy, AFM, XPS, TEM, XRD	[87]
	PCL	PCL/MMT	53.17 °C	Nil	Nil	HNMR, TGA, FTIR, XRD, TEM,	[60]
	MWCNT-FA	SBR	150 °C	90%	100 °C, 5 h	FTIR, TGA, DMA, Raman spectroscopy,	[49]
Quadruple Hydrogen Bonding	PU	PU-AHMP	<−20 °C	97%	25 °C, 1440 min	ATIR, TGA, DMA, DSC, SST	[63]
	Fe3O4	P(MA-co-UPyMA)	−2 °C	Nil	40 °C	TGA, FTIR, TEM, DSC	[65]
	POSS	DDSQ[P(BA-co-UPyA)]2	−48 °C	Nil	40 °C, 15 h	TEM, FTIR, DSC	[66]
	POSS	P(DDSQ-COD-co-UPy)	−11 °C	100%	25 °C, 24 h	TGA, HNMR, GPC, TEM	[88]
	SiO_2_	SiO2-UPy	−1.8 °C	Nil	Nil	TGA, DSC, HNMR, FTIR SEM, SST, DMA	[64]
	PPy	PPy/PEG–UPy	Nil	100%	25 °C, 5 min	TGA, HNMR, SEM, FTIR, SST	[89]
	rGO	PAA-rGO	Nil	95%	25 °C, 30 s	FTIR, SST, SEM	[90]
π−π interactions	CNC	Ph-NDI	Nil	90%	85 °C, 30 min	SEM, TEM, TGA, SSM	[91]
	P-AuNPs	Py-PDA-P-AuNPs	−7 °C	108%	−50 °C, 10 min	UV, TGA, DSC, EDX, SST	[71]
	rGO	PS, PSCN, PSNP	14.3 °C, 4.4 °C and 25.2 °C	Nil	Nil	TGA, NMR, SEM, DSC, Raman, TEM	[72]
	Si-GO	PU	Nil	100%	50–60 s, 459 W	TGA, DSC, FTIR, Raman, PXRD, EDX	[73]
Metal–Ligand Interaction	MWCNT	Zn^2+^-CNT/PU	Nil	97%	90 °C	DSC, TGA, FTIR, SP, SST, XRD, HNMR, TEM	[78]
	CNT	CNTsx-g-CPy/Zn	0–80 °C	93.6%	4 h	HNMR, SEM, TGA, FTIR, SST	[80]
	Fe_3_O_4_	Fe_3_O_4_x-g-CPy/Zn	10–100 °C	96.2%	70 °C, 8 h	HNMR, TEM, TGA, SST, SEM	[81]
	SQ	MI/MT-SiO_1.5_	−29 °C	80%	50 °C, 24 h	HNMR, TGA, DSC, XRD, SEC, SFM	[79]

## 4. Shape Memory Polymer (SMP) Nanocomposite 

SMP nanocomposites represent a promising class of polymer nanocomposites with a wide range of applications. By incorporating various types of nanofillers into the polymer matrix, the properties of the material can be altered, allowing for tailored properties and desired capabilities. CNTs have proved to be a promising candidate for use in SMP nanocomposite because they provide a better hybridization and synergistic effect between polymer and nanofiller compared to other nanocomposites. 

CNTs

The use of electrical energy for the manipulation of polymer nanocomposite offers numerous benefits, including a high degree of shape recovery ability for various electro-stimulated applications. SMPs fabricated via electrical actuation result in numerous advantages for producing a high-level performance electro-active system. The addition of CNTs to a 3D polymer acts as a conducive network and improves the mechanical properties. The addition of CNT into the polymer increases the storage modulus over a diverse range of temperatures, enhancing from 3.0 GPa to 2.4 GPa with the addition of nanofillers. The flexural stress during shape recovery was calculated to be 14.6 MPa. Adding CNT to the matrix also enhanced T_g_ from 90 °C to 100 °C, and upon applying voltage, the polymer nanocomposite responded in 8 s due to the 3D CNT network’s random charge distribution [92]. A polymer system comprised of polyhydroxyethylmethacrylate (PHEMA) and MWCNT showed electrical conductivity and remote heating. The polymer showed a heat-triggered healing response. The presence of HB in the matrix increased T_g_ and thermomechanical properties by restricting chain movement, and these increased with a growing constituency of CNT. This property enhanced the electroactive property, with a lower voltage requirement. Electrical heating enhanced the shape fixity and shape recovery via the joule effect, reaching maximum values of 100% and 95%, respectively [93]. SMPs with hybrid MWCNTs and montmorillonite nanoclay (MMT) showed electrical and thermally induced recovery. However, electrical-induced recovery of the polymer nanocomposite presented a faster trend because the nanofiller exhibited a greater impact on thermal actuation. However, recovery time is shorter in the electrically modulated recovery, with a ten-times faster rate than thermal activation. In thermal activation, the polymer nanocomposite achieved 80% recovery in 250 s, while electrically this recovery was reached in 30–35 s [94]. PU comprised of MWCNTs and halloysite nanotubes (HNTs) provided 4D-printed polymer nanocomposite with a thermally stimulated shape memory ability. HNTs augment the healing of the polymer with MWCNTs by 34% and impart a high flexural strength of 57%. The polymer presented a shape memory recovery of 70% in only 120 s. Increases in the percentage of MWCNTs enhance the recovery ability. Nanofillers in the polymer system improved the crystallinity by acting as nucleating agents, which increased the cross-linking density, resulting in increasing the T_g_ [95].

Graphene

**Figure 6:**
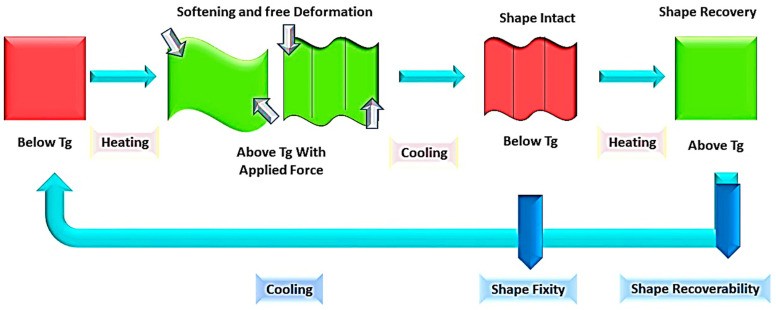
Shape memory behavior and recovery of polymer nanocomposites.

Graphene-based nanomaterials are very efficient in producing shape memory polymer nanomaterials possessing outstanding shape recovery and improved glass transition temperature (Figure 6). A poly(vinyl alcohol) polymer matrix with GO and rGo as nanofillers demonstrated that the addition of GO and rGO to the resin increased the T_g_ from 81.10 °C to 106.50 °C and 101.15 °C, respectively, due to the strong interface interaction between the nanofillers and the polymer matrix. The bending test revealed that the nanocomposite exhibited excellent mechanical properties, with shape recovery and shape fixity values up to 95%. This high degree of recovery fixity is due to the presence of greater crystallinity and higher HB [96]. An SMP nanocomposite composed of polycaprolactone/thermoplastic starch/graphene nanoplatelets (PCL/TPS/GNPs) provided a desirable healing ability by adding GNPs. The polymer exhibited a 70% shape recovery and a 90% shape fixity ability. The position of GNPs on the starch phase increased the healing ability of the material; however, increasing GNPs lowers the ability because of agglomeration. The presence of glycerol increases the shape recovery ability in water stimulus [97]. A cryo-system-assisted extrusion method for the fabrication of a 3D epoxy polymer nanocomposite by adding graphene nanoplatelets provided near set shape SMPs. The GNPs increased the tensile strength of the system by 30%, along with a 17% increase in the elasticity of the 3D-printed composite. Compared to the pure polymer nanocomposite, the SMP showed an increase of 365% in the storage modulus [98]. SMPs with GO and CNT nanofillers combined show remarkable electric and mechanical properties. In the polymer matrix, CNTs work to bridge the crack of the polymer while GO deflects the crack. These nanofillers improve the interfacial loading, which improves the elastic modulus of the polymer system by 32%, with a storage modulus increase of 40% at room temperature. A good dispersion of the nanofillers reduces the damping ability of polymer chains, inhibiting chain mobility and improving mechanical properties [99]. 

Other fillers

In other research works, the addition of magnetic nanomaterials to nanofillers has improved the shape recovery ability, and magnetic nanofillers induce superparamagnetic properties to the polymer matrix. In a recent study, bio-based benzoxazine resin infused with Fe_3_O_4_ nanofiller induced healing ability actuated by light and magnetic fields. The infusion of the nanofiller into the benzoxazine/epoxy copolymer swelled the fixity value from 85% to 93%. The shape recovery of the polymer reached 98%, while recovery stimulated by the magnetic field reached 99% in 26 s. The actuated shape recovery of magnetic fields makes them a good candidate for soft robotics [100]. 

Controlled concentration is a key factor for the effectiveness of some nanomaterials, as their content can decrease the shape repairability. Silica nanoparticles, for example, have been found to reduce both shape fixity and self-healing when their constituency increases from a certain level. In a study by Choong et al., it was reported that the infusion of silica nanoparticles into the polymer matrix improves the mechanical strength by forming multifunctional crosslinking, hence increasing the R_f_ to 100% by increasing the nanosilica concentration. However, by increasing nanosilica concentration, the shape recovery value decreased [101]. In another study, it was reported that the infusion of SiO_2_ nanoparticles into a blend of soft and hard polymer provided remarkable R_f_ and recovery values. The R_f_ value increased from 93.03% to 100% and recovery value increased from 90% to 100% [102].

A polydopamine nanofiller in a polymer nanocomposite comprising a PCL/TPU blend caused light-actuated recovery. The addition of polydopamine led to light-induced shape fixity, with 100% shape recovery. The matrix showed light-induced healing within 150 s, with a healing efficiency of 78.53%. An increased content of PCL in the polymer nanocomposite enhanced shape recovery and shape fixing ability. The incorporation of PDA into the polymer blend resulted in the enhancement of shape memory by up to 100% within only 10 s of 500 mW/visible light irradiation [103]. The incorporation of cellulose CNTs induces a water-responsive sensing ability in polymeric material. PU with a disulfide bond and CNTs produces a flexible photonic polymer material that makes a frame outside the chiral nematic structure, helping to make it impossible to collapse upon multi-stimulus response. This outstanding ability enhances the mechanical properties with multi-cycle reproducibility. The S–S bond imparted toughness to the film and showed that 500 g of polymer could hold up to 5500 times its own weight [104].

### 4.1. Thermo-Responsive Shape Memory Polymer (TRSMPs) Nanocomposite and Their Mechanical Properties 

The recovery of polymers is mediated via various stimuli that activate the recovery of the resin from a temporary to a permanent state. These stimuli include electric charge, magnetic field, hot water, infrared light, or visible light (Figure 6). For instance, thermos-responsive polymers can recover their shape though the application of heat. Nanomaterials are often added to the polymer matrix as fillers to enhance mechanical strength, glass transition temperature, and mechanical properties.

**Figure 7:**
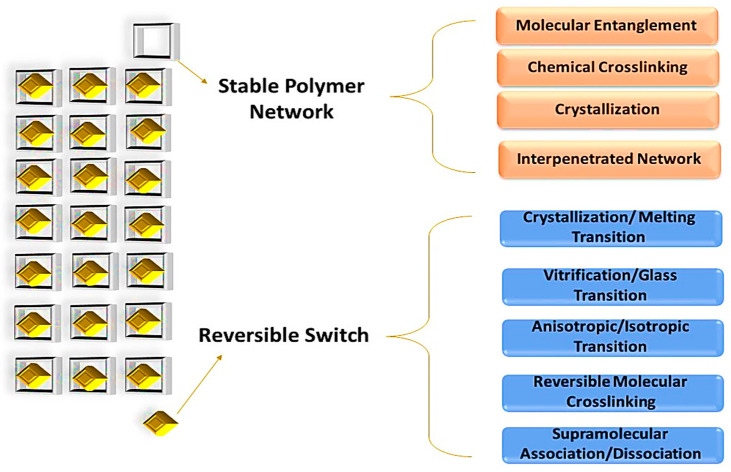
Different molecular structures of SMPs and different mechanisms that are observed in the SME.

**Figure 8:**
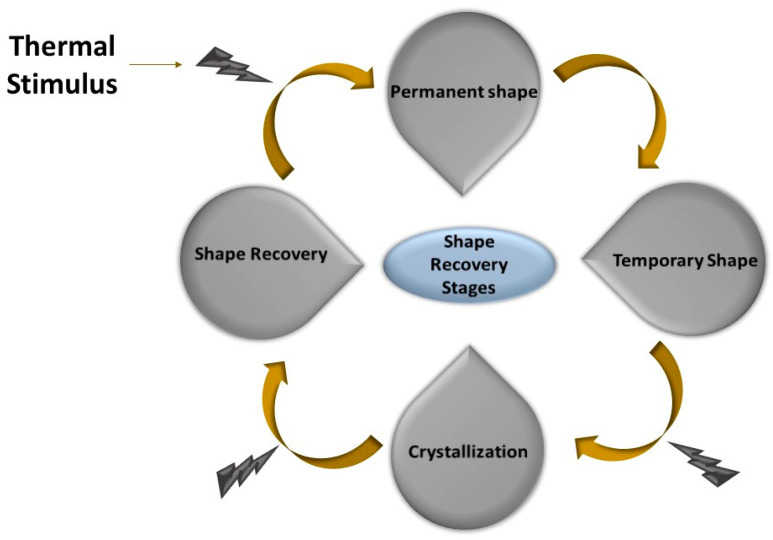
Shape recovery under heat stimulus.

The mechanical properties and shape recovery is dependent on the nature of the additives used for reinforcement following varied mechanism (Figure 7). TRSMP nanocomposites based on a PCL styrene-butadiene rubber polymer matrix with nickel oxide, iron oxide, and iron–nickel oxide nanoparticles show distinct inbuild properties. The T_g_ of the polymeric blend was 64 °C, and shape memory ability depended on the PCL melting temperature. A temperature higher than the transition temperature resulted in a fast shape recovery due to the melting of soft segments. The polymer with Fe_2_NiO_4_ took the longest time to recover because of hardness and brittleness, which also increased the Young’s modulus. Meanwhile, the polymer with Fe_2_O_3_ showed a better elastic and stress-retaining ability, and the mechanical abilities were increased. The resins were recovered in hot water at 75 °C within seconds, with a recovery ability of 90%. The shape recovery ability, along with the shape fixity ability, of SMPS-Fe_2_O_3_ was higher than the other nanocomposites, with the lowest recovery time [105]. TRSMP nanocomposites with recovery and fixity temperatures close to body temperature have been reported in several studies, making them highly useful in biomedical materials, breathable clothing, and other applications. The addition of polydimethylsiloxane (PDMS) in PCL influences the mechanical properties so much so that the resultant polymer adjusts the melting temperature. The triggered temperature of the polymer showed a near body temperature of 37 °C, caused by PCL–PDMS arrangements. Increasing the concentration of PDMS enhanced the tensile strength from 1.41 to 6.64 MPa, while elongation increased from 122.7% to 154.4%. However, the Young’s modulus reduced from 12.65 MPa to 3.42 MPa. After successive cycles, the film showed a shape fixity of 98% and a shape recovery of over 100% due to HB and dipole interaction [106]. A triple responsive polymer composed of polyolefin, lauric acid, and carbon black nanoparticles (POE/LA/CB) exhibited a stimulus response towards three stimuli including solvent, electrical, and thermal responses. In the polymer matrix, CB played a crucial role as increasing its concentration decreased the crystallization temperature, and the addition of CB reduced the strain and increased resistance towards external forces. POE/LA/CB (50/50/20) showed an almost complete shape fixity of 96% and shape recovery of 97% at 65 °C in only 10 s for multiple cycles, without any decline in the polymeric properties. The storage modulus of the polymeric content increased to 300%, with greater stress recovery, while elongation at breakage reached 310%. CB also increased the electrical conductivity of the polymer, with ohmic resistance reaching ~23 Ω [107]. A thermoresponsive polymer with poly(L-lactide) (PLLA), PU, and CNT also showed a similar trend, with CNT illustrating a larger influence on the rheological properties. A temperature of 75 °C was required for shape recovery. High temperature and increasing CNT content also restricted its ability by an increased percolation network [108]. The GO/rGO hybrid as a nanofiller in the resin exhibited better and more homogenous dispersion in the resin than the other nanofillers. GO content increased the T_g_ and reduced chain movement, while rGO slightly reduced it. The rGO interacted with soft segments, with a low impact on melting temperature. The hybrid depicted an easy tunable ability and exhibited the best heat-mediated shape recovery (Figure 8) of 96.7% and shape fixation of 99.1% [109]. TRSMPs are comprised of poly(styrene-b-isoprene-b-styrene) (SIS) and poly(ethylene-co-1-octene) (PEO). The shape memory study of the polymeric blend showed that 60PEO/40SIS exhibited the maximum shape fixity of 95.74% and shape recovery of 98.98%. The melting and crystallinity temperature of PEO had minimal effects on the presence of SIS. However, crystallinity decreased with increasing SIS, owing to the interaction between the PI block of the SIS and the PE chain of the PEO. The T_g_ of the blend was reported to be 75 °C. Temporary shape fixity and recovery occurred at −20 °C when crystallization of the PEO was completed [110]. TRSMPs with PU, MWCNTs, and HNTs showed significantly improved mechanical abilities and recovery modulus. The specimen under investigation showed 100% shape recovery with MWCNT reinforcement in 105 s, but the polymer with HNTs showed 80% recovery. The strength of the polymer increased by both HNTs and MWCNTs. As the content on the fillers increased, the impact energy of the TRSMPs increased by 250% [111]. TRSMPs based on maleic anhydride-grafted polystyrene-block-polyethylenebutylene-block-polystyrene-triblock copolymer (MA-g-SEBS) and cellulose nanofibrils (NCs) as reinforcement provide amplified tensile strength, storage modulus, and elongation at breakage via improved interaction between the NCs. This helps in aligning the cellulose fibrils and copolymer chain parallelly, which make a orientated composite. This orientated composite showed an enhanced thermal mediated recovery (hot water at 75 °C) of 92%. The acrylation of NCs increased the R_f_ of the TRSMPs to 94%. The polymer also demonstrated good reproducibility, making them a good candidate for biomedical applications [112]. Three-dimensional-printed PLA/PCL-based TRSMPs with cellulose nanocrystal-organic montmorillonite (CNC-OMMT) provide a versatile shape recovery ability of 99% at 65 °C. Increasing the nanofiller constituent reduces the recovery ability due to hindrance in chain mobility. The nanofiller increased the flexural strength, tensile strength, and notched impact strength by 37.1%, 25.8%, and 24.2%, respectively [113].

### 4.2. Light-Actuated Shape Memory Polymer Nanocomposite

Light is considered as the ideal candidate for the induction of shape memory response in polymer nanocomposites because it is more environmentally friendly, induces quick response, and can be controlled remotely. Creating a polymer nanocomposite exhibiting a light-mediated response is more direct because the addition of fillers can induce this behavior. Light-induced recovery response depicts a small area of action, which makes it more suitable for the recovery of specific probes than heat-induced recovery.

An SMP blend composed of PDA, PCL, and TPU is the blend type that depicts light-actuated behavior. The addition of PDA imparts a trend in the polymeric blend of PCL and TPU. The polymer nanocomposite showed a faster light-mediated response. Under visible light, the polymer was recovered in 10 s, and increasing the content of PDA increased this behavior. In 50 s, the Rf value of the polymer reached 100%. The PDA acted as the medium that transformed light energy into heat, recovering the shape. The polymer blend worked well under a light intensity of 500 mW/cm^2^ [103]. Similarly, the addition of PDA to polynanoborane (PNB) also depicted light-induced shape memory recovery in the NIR region. The recovery ability of the resin was excellent as it reached its original shape (99.9%) in 20 s under an infrared light of 2.5 w/cm^2^. Apart from the light-mediated recovery, the polymer also showed a thermal-induced recovery response [114]. 

In another study, a novel polymer nanocomposite was prepared by inducing copper sulfide (CuS) nanoparticles in the PU blend. CuS nanoparticles triggered a photo-induced shape memory ability in the NIR region. Healing efficiency was dependent on the concentration of the CuS content. An amount of 0.05 wt% of the nanoparticles induced 78.6% shape memory ability, but as the content increased to 0.1 wt% the ability increased to 99%. The addition of inorganic nanofiller also enhanced other mechanical properties. With 0.1 wt%, the elongation at breakage depicted a value of 1102.3%, while the stress-retaining ability of the polymer film was 21.54 MPa [115]. A scaffold SMP with poly(n, L-lactic acid-co-trimethylene carbonate) (PLMC) with modified GO (g-GO) and silica nanoparticles gave a polymer nanocomposite that depicted shape memory behavior actuated by NIR light. The g-GO helped in building the scaffold structure and induced a faster shape ability in 8 s. Under NIR light, four corners of the structure were erected, which increased the healing ability of the material [116]. CNTs are also used as photo-responsive agents in the polymer network to induce shape recovery of the polymer material. In the polymer resin of PVA and pyrene, CNTs impart an ultrafast recovery response by HB interaction and stacking. The recovery time of the material was 98% in 10 s under an NIR light of 150 MW/cm^2^, while the recovery time of the spiral material was 30 s [117]. Polypyrrol (PPy) is another material that imparts a photo-responsive ability in polymer networks. The addition of PPy to the blend of PLA and PCL provides a triple shape memory recovery ability. The polymer blend exhibited a triple recovery ability of 92%, 87%, and 93%, while the addition of PPy enhanced the recovery ability to 95% under NIR light. PPy recovered the polymer remotely, and with accuracy. The polymer also showed a self-healing ability of 90% [118].

### 4.3. Magnetically Actuated Shape Memory Polymer Nanocomposite

The addition of magnetic nanofiller to the polymer nanocomposite imparts a shape recovery ability to the polymer blend that is responsive towards magnetic fields to increase the heat of the probe, causing healing. The energy loss of the magnetic nanofillers used in the polymer blend stimulates the generation of heat. Heat in the polymer blend is generate by the loss of high-frequency magnetic field nanomaterials [119].

The addition of Fe_3_O_4_ and CNTs simultaneously in the PU exhibits ferrimagnetism, with a saturation magnetization of 4.6 emu.g^−1^. The polymer demonstrated excellent recovery ability, with 10% CNT/ Fe_3_O_4_. Under a magnetic field of 50 Hz, 4 kAm^−1^, the polymer showed a shape memory recovery of 64.6%. The addition of nanofillers to PU did not change its shape fixity ability. Decorated CNTs enhanced the stress recovery by 110% [120]. Utilizing Fe_3_O_4_ in biobased benzoaxine with an epoxy polymer imparted a magnetic response, causing shape recovery stimulated by the magnetic field. Under a magnetic field of 750 to 1100 kHz, a polymer infused with 5%wt Fe_3_O_4_ was recovered from 80 to 99% in only 26 s [100]. An Fe_3_O_4_-grafted copolymer, Fe_3_O_4_-*g*-P(TMA-*co*-LA-*co*-VI), further coordinated with Zn, also showed magnetic field-induced shape recovery. The polymer demonstrated 300% strain breaking and 1.66 MPa of maximum stress. Increasing the content of Fe_3_O_4_ in the polymer blend increased the magnetization. Fe_3_O_4_ also imparted an outstanding magneto-caloric response under varied magnetic fields. Under a small magnetic field, the temperature of the resin rose, and as the magnetic field diminished it dropped to room temperature in only 200 s, which stimulated the shape recovery. In 60 s, the polymer under this magnetic field recovered its original shape completely [81]. Three-dimensional printing of cellulose nano-fibrils and Fe_3_O_4_ in the poly-hydroxybutyrate/poly(ε-caprolactone) blend demonstrated a magneto-responsive shape memory behavior. A hot-pressed probe showed a shape fixity of 100%. A 15% content of the nanofiller led to recovery in 20 s due to increased heat induction on the application of the magnetic field. However, in the hot-pressed probe, the recovery time is further reduced to only 8 s, which is attributed to the chain hindrance of cellulose nanofibrils. The polymer film showed a tensile strength of 60.67 MPa, demonstrating an increase of 55.13% compared to the hot-pressed film, which had a tensile strength of 39.11 MPa [121]. HNTs, along with Fe_3_O_4_, in the PU matrix improve the shape recovery ability, giving the highest recovery of 179 GPa·°C^−1^. The polymer nanocomposite exhibited the highest shape stability of 96.7%. This high degree of shape recovery is because adding CNTs to the polymer nanocomposite enhanced the amount of soft segments. An amount of 2 %wt of HNTs induces a larger shape recovery ability under magnetic fields [122]. Four-dimensional-printed PLA and TPU resin with Fe_3_O_4_ showed a high recovery response of 96.4% in 40 s under magnetic fields. This high response is due to the high heat generated by magnetic nanoparticles. The polymer infused with magnetic nanofillers demonstrates a shape fixity of 100%. Increasing the content of Fe_3_O_4_ accelerates the shape recovery ability [123].

### 4.4. Electrically Actuated Shape Memory Polymer Nanocomposite

In the electric field-stimulated shape recovery of SMPs, the polymer nanocomposite is exposed to an electric field, which triggers the shape recovery. Pristine polymers are not conducive to electric fields, which is why electrically active fillers are added to the polymer matrix to reduce the electrical resistivity. One such filler that is added to the polymer blend is CNTs, which improve its electrical conductivity and induce shape recovery by the electric field. In the epoxy polymer, CNTs are added, which improves its mechanical properties by interfacial interactions. The elastic modulus of the polymer nanocomposite enhanced from 52% to 514% for temperatures of 20–50 °C. Similarly, in the temperature range of 30–90 °C the storage modulus also increased from 60% to 82%. Under an electric field of 12 V DC, the polymer demonstrated a shape recovery ability of 180° deformation in 22 s [124]. In another study, CNTs with a polyketone polymer also depicted a remarkable healing ability. Under an electric field, the polymer nanocomposite shows a network rearrangement, which is caused by the relaxation of the polymer chain at higher temperatures than T_g_. An electric field is applied in the range of 50–60 V. At 40 V, the specimen demonstrated a shape recovery ratio close to 0.9, with a recovery rate of 30 to 80 mrad/s. However, prolonged heating causes resistivity in the polymer, leading to deformation [125]. Adding graphene to the epoxy polymer also enhances the electrical stimulus. Increasing graphene content enlarges the electrically actuated shape memory ability of the polymer nanocomposite. A 0.4% graphene content in the matrix provide shape memory properties of 180 S, 140 S, and 95 S under electric fields of 40 V, 60 V, and 80 V, respectively [126]. A blend of PCL with maleic anhydride-grafted polystyrene-block-poly(ethylene-co-butylene)-block-polystyrene (SEBS-g-MA) and MWCNTs (PCL/SEBS-g-MA/MWCNT) also provides an electrically active polymer nanocomposite. The polymer nanocomposite showed a small recovery time of 56 s at 40 V, with a recovery ratio of 87.97% and shape fixing ability of 98.28%. With a ratio of 50/50-5CNT, the recovery reached 93.33% in 390 s at 100 V, and a recovery ratio of 50/50-10CNT reached 91.7% at a lower voltage (40 V) in a shorter time (56 s) [127]. PU induced with CB as a nanofiller exhibited high T_g_. Under DC 60 V, the matrix demonstrated a 98% shape recovery. Twenty-five percent of the CB content in the polymer matrix exhibited enhanced electrical and mechanical properties. The shape recovery is due to joule heating, which is triggered by the electrical stimulus [128].

## 5. Biomedical Applications 

**Figure 9:**
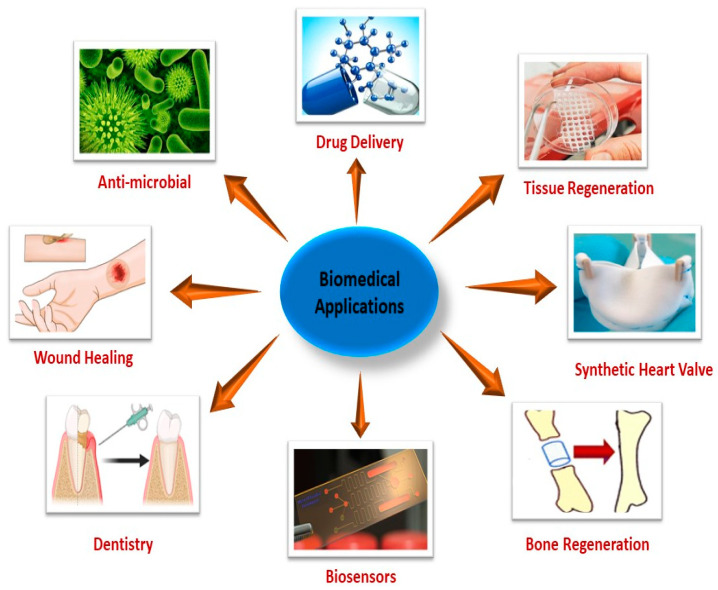
Biomedical applications of SHPs and SMPs.

SMPs and SHPs can be utilized in several biomedical applications owing to their healing ability (Figure 9). They have been reported by Lendlein et al. as being used as self-tightening sutures, which work at a physiological temperature of 41 °C [129]. Their ability was further investigated by Biswas et al., who reported that the self-tightening ability of the stent was enhanced after 11 s of being placed inside the human body [130]. Apart from that, they also have applications in drug delivery, orthodontics, tissue engineering, orthopedics, and endovascular surgery. As the human body has an optimal operational range, switching the polymer to the above, which is lower to that range, helps in the optimization of the material in order to have the best efficiency. This can be done by the remote triggering of the material, which can be induced by ultrasound, infrared rays, lasers, etc. [131,132]. Aliphatic polyester polymers have applications in wound healing and implants because of their manageable mechanical and physical properties [133]. In endoscopy, wound healing is a major problem, but Lendlein and Langer utilized smart thermoplastics comprised of oligo(ɛ-caprolactone) diol, which shrunk the wound at body temperature [129]. In irregular bone shape, memory polymers also have tremendous applications. Zhang et al. utilized a PCL diacrylate-based shape memory polymer to modulate the bone defect. For this purpose, a temperature of 55 °C was applied, which causes the softening of scaffolding upon application of force. After the force removal the scaffold expands, and as it cools the scaffold self-fits into the defects [134].

SMPs are also used for cardiovascular diseases. In the case of aneurysms, a thermal responsive PU is positioned in the aneurysm sac. By means of thermal stimulus, the cavity fills, showing only slight inflammatory response with proved biocompatibility [135]. PLLA and poly(D-lactide) (PDLA) are broadly used as scaffolding materials in the regeneration of bonds owing to their slow degradation, biocompatibility, and tensile strength, but their properties are enhanced by the use of various nanofillers [136]. POSS with PLA form a macroporous composite and exhibit the ability to release recombinant osteogenic bone morphogenetic protein-2 and support osteogenesis and cell attachment, which help in the formation of next-generation artificial bone grafts [137]. PDA-induced PCLs show good biocompatibility with NIR-induced memory recovery, demonstrating the potential to be used as biomedical implants [138]. Thermoresponsive polymers based on bis-acrylamide-nucleic acid duplexes/boronate ester-glucosamine-nucleic acid duplexes and aurum nanoparticles as fillers demonstrate a good shape memory effect by permanent interfacial interaction and healing via nucleic acid hybridization, which aid in regaining stiffness and promoting drug release [139].

PC-based PU also has applications in orthodontics. Polymeric wire is stretched by applying temperature and is then attached to the bracket and heated, which triggers shape recovery, causing teeth alignment [140].

SHPs comprised of selenium have distinctive properties, which include drug-releasing systems and excellent anti-carcinogenic activity achieved by the combination of both photo- and chemotherapy. Exposure of cancer cells to γ-radiation causes apoptosis; however, in the treatment, selenium-based polymers respond as a controlled release system towards γ-radiation, with controlled chemotherapy giving better results in cancer treatment [141].

The printing of polymers for tissue regeneration and transformation has also shown promising results, and may provide a solution for organ or tissue shortages. These synthetic tissues are used as tissue replacements and drug-delivery agents in surgeries. These synthetic cells also provide an electrical connection for the damaged nerves [142]. The polymer consists of PEI and PAA and shows dual drug delivery properties, with drug release followed by the surface-activated method. Diffusion of the polymer filament and ionic change activate the healing ability, along with regaining drug delivery and loading ability [143]. The regenerative and cryopreservative ability of mesenchymal stem cells makes them useful for the wound healing process cases of major conditions such as spinal cord injury [144]. PMMA incorporated with pins and screws can be used for the healing of cervical cracks in the skull base of dogs. PMMAs showed a considerable regenerative ability in skull healing, which make them a potential candidate for bone engineering [145].

SHPs are also used as artificial human skin. Polymers made of PDMAA-PVA infused with rGO exhibited a ten-times better tensile strength than human skin, high conductivity due to rGO, thermal stability, and biocompatibility. The polymer also showed touch sensitivity and generated electricity on the application of pressure, facilitating healing abilities [146].

SHPs are also used in dentistry. Resin composed of amorphous calcium sulphate nanoparticles and induced with dimethylaminohexadecyl methacrylate (DMAHDM) microcapsules have the ability to recover track up to 67%, with a high anti-film ability. The film also demonstrated an anti-bacterial ability and calcium delivery ability, along with remineralization of the crack [147]. Dentine bonding made of PU encapsulated by TEGMA displayed a healing ability of 90.5%, with zero cytotoxicity. The self-healing dentine covering heals the crack before the formation of bacterial growth and shows a high tensile strength of 25.8 MPa [148].

Self-adaptive polymers can also be used as sensors and actuators that change their shape and physical properties upon different stimuli. Lee et al. reported on a hydrogel actuator that utilized metal coordination and ionoprinting chemistry and was operated via the pH change [149,150]. As sensors, these polymers and hydrogels can be used as flexible sensors to detect human motion [151,152]. SHP nanocomposites are comprised of PVA and PAA with Au filler nanoparticles and show a photothermal self-healing function, with an effective anti-bacterial ability of almost 80%, with wound healing ability [153].

## 6. Other Applications

### 6.1. Aerospace

The low weight of SMPs and SHPs make them the best candidates for the aerospace sector. Their healing ability makes them the best choice for use as hinges, space deployable structures, and morphing aircraft. SHPS can be used as spacecraft coatings. SHP-based high-strength alloys are also used in the aerospace industry [154]. SMPs are used in morphing in aircraft. Because of their shape recovery ability, smooth deformation is seen, which is achieved in 98 s of the application of stimulus [155].

### 6.2. Textile

In textiles, SMPs add fascinating properties, leading to smart textiles. They are used to make breathable fabric, which has the capability of transmitting vapors of water from the body surface to its surroundings, along with the ability to protect from wind and rain. Breathability in the fabric is achieved by coating the surface of the fabric with laminated microporous film [156].

SHPs in various textiles enhance the efficacy of the product. Fabrics treated with reinforcement show larger stability. The use of methyl vinyl silicon along with silsesquioxane on rubber gloves enhances the stability of the material [157]. Similarly, self-healing electromagnetic interference shielding fabrics have also been developed [158].

### 6.3. Sensors, Electrical, and Electronics

SHPs and SMPs are also used in energy harvesting devices to contribute huge energy needs as flexible devices and electronic devices. PPy-based hydrogels are used as sensors because they exhibit outstanding resistance and remarkable stretching ability, which makes them good specimens for bending and compression motion [159]. SMP-based triboelectric nanogenerators are not only used for energy harvesting but are also utilized as biomechanical motion sensors [160]. SMPs are also used as information energy storage devices. Xie et al. [161] combined photo-imaging and shape memory information into one system, which produced a 3D signal and a photoimaging 2D signal that would be visible only in UV light, making the system an anti-counterfeit one.

### 6.4. Paints and Self-Healing Coatings

Epoxies are used in paints and coatings and enhance the appearance of the materials. They also have healing ability. The modification of the epoxy using various additives enhances its healing ability, along with its mechanical properties. Combining GO with epoxy enhances its healing ability by the achievement of stiffness. The hybrid LbL assembling of GO with PVA and tannic acid greatly enhance its stiffness and hardness [162]. Introducing TiO_2_ to the epoxy also enhances the anticorrosive ability of the epoxy polymer. The damage to the coating healed in 96 h with the enhanced anti-corrosive ability in saline water [44]. UV-responsive coatings have also been formulated and demonstrate a self-healing ability under UV stimulus. PPG-PDMS-Zn-0.5 demonstrates a self-healing ability of 97% at −20 °C, with a tensile strength of 0.98 MPa [163].

### 6.5. Construction Materials

SMPs and SHPs are used in construction materials due to their durability and strength. Poly(ethylene-co-acrylic acid) zinc salt powder, bisphenol A diglycidyl ether (BPA) is used as a polymer cement composite with a self-healing property at 300 °C for 30 days. The polymer composite showed a remarkable strength of 1000 psi after the passage of one day [164]. Polymers can also be used as self-sealable materials. Sulfur polymer mixed with Portland cement and calcium sulfo-aluminate gave a binary cement that shows a super absorbent ability and heals damage in 30 min [165].

## 7. Conclusions

Shape memory polymers (SMPs) and shape memory polymer nanocomposites exhibit a wide range of mechanical properties, depending on the specific polymer materials employed and their respective ratios. In the case of SMP nanocomposites, various mechanisms, including the Diels–Alder reaction, thermal energy, quadruple hydrogen bonding, π–π stacking interactions, and ligand–metal interactions, contribute to their mechanical properties. Among these mechanisms, quadruple hydrogen bonding stands out for its applicability in biomedical and various other fields due to its ability to heal at or near room temperature. Conversely, shape memory polymer nanocomposites typically rely on thermal and electrical stimulation for healing, demonstrating rapid shape recovery within seconds and maintaining superior mechanical properties, even through multiple cyclic processes.

## 8. Future Perspective

Shape memory and SHP nanocomposites have found their applications across a wide range of research fields and societal domains. Several approaches have been explored to produce high-quality polymer nanocomposites with exceptional mechanical properties and faster healing mechanisms, but the advancements continue to evolve. Although different mechanisms have been proposed for the favorable material, the majority require external forces for the healing process. Therefore, efforts are underway to develop materials with healing mechanisms that are independent of external forces. However, some reactions do not produce materials with high mechanical properties, which is a prerequisite for shape memory and SHP nanocomposites. The exact mechanism of action and behavior of self-healing and shape memory polymer nanocomposites are still unknown and need to be studied further. In addition, there is a need to develop polymer nanocomposites with an automatic intelligent system to ward off damage without harming or damaging the product.

Over the years, two strategies have been developed to repair damage on both microscopic and macroscopic levels: shape memory-assisted self-healing (SMASH) and close then heal (CTH). SMAS involves the closure of crack followed by the polymer healing itself, while CTH also involves crack closure but requires a healing agent or dispersed micro-capsules for the polymer healing [166]. They both have applications in the electrical, medicinal, aerospace, textile, and robotics fields, with prospective uses in various other fields. These materials, being light weight, are promising for use in paneling, reflectors, solar panels, booming, and so on. These polymers are less toxic, biodegradable, and recyclable, which make them useful for medical applications such as drug delivery, bone fracture fixing devices, bone engineering, intervascular valves, dentistry coatings, skeletal fillers, and other applications. These materials, because of their healing ability, have a promising future for the automative field in making self-healing tires, tunable car structures, developing seat assemblies, and robotics. SHPs and SMPs can also be used for self-healable adhesives for coating. In textiles, they are used for breathable and waterproof clothing. In the electrical field, they are used for sensors, actuators, microfluidic structures, soft electronic toys, and more. The incorporation of different nanofillers into SHPs and SMPs enhances the activities and application of the polymeric materials significantly. This enhancement in applications represents vast future perspectives in almost every sphere of life because the scientific community in this field is interested in advancing the development of polymer nanocomposites with multipurpose mechanical and healing abilities for wider tolerance towards potential damage, and significant robustness. The emergence of new materials with such improved healing abilities and wider applications is a promising step in achieving that goal.

However, there are expectations from researchers that they will contribute their fair share in studying further applications in this field. Smart application of these materials in the field of electronics are expected to increase with enhanced self-healing abilities. Owing to the growing energy demand, it is also expected that, in future, more focus is given to novel polymer nanocomposites with more flexibility, durability, mechanical strength, and a larger share of energy in the main energy grid.

Currently, the formation of different polymer nanocomposites is more complex. It is expected that, in future, additional simpler approaches will be attained, maintaining or improving the advantages of these materials. It is also expected that such SMPs and SHPs will be fabricated with the ability to recover remotely under remote stimulus, along with improved healing times and efficiency of the material.

## Data Availability

Not applicable.

## Abbreviations

Multiwalled carbon nanotubes (MWCNT), furan-derived PK30 and the aromatic bismaleimide (PK30xFUyA2Pz/B-Ma/MWCNT), Graphene nanoplatelets (GNPs), Furfuryl derivatives (FD), Polyurethane (PU), heat healed (H-H), IR healed (IR-H), microwave healed (MW-H), furfuryl amine and 6-Maleimidocaproic acid (FGE-EDR/MDPB@FGO), graphene oxide (GO), graphene oxide material (rmGO), polyurethane-thermal melamine with-graphene oxide material (PU-EDM/rmGO), iron oxide nanoparticle-decorated multiwalled carbon nanotubes (IONPs-MWCNTs), polycaprolactone-poly(furfuryl glycidyl ether) copolymer-1,10-(methylenedi-4,1-phenylene)bismaleimide (BMI)-(IONPs-MWCNTs), mGO (maleimide functionalized graphene oxide), mechanical stress–strain mechanism (MSSM), halloysite nanotube (HNT), bismaleimide joined graphite nanoplatelets (B-GNPs), furfuryl functionalized polyurethane(mFPU), reduced graphene oxide grafted magnetite nanoparticles (rGO/Fe3O4), poly(ε-caprolactone) (PCL), montmorillonite (MMT), styrene butadiene (SBR), furfuryl functionalized MWCNT (MWCNT-FA), static strain test (SST), 2-amino-4-hydroxy-6-methylpyrimidineP with Polyurethane (U-AHMP), 2-ureido-4[1H]-pyrimidinone methyl methacrylate (P(MA-co-UPyMA)), polyhedral oligomeric silsesquioxanes (POSS), n-butyl acrylate (BA) with 2-ureido-4[1H]-pyrimidinone acrylate-POSS (P(BA-co-UPyA)s), 3,13-dihydrooctaphenyl double-decker silsesquioxane—cyclooctadiene(P(DDSQ-COD-co-UPy), polypyrrole doped Poly 2-ureido-4[1H]-pyrimidinone (PPy/PEG–UPy), polyacrylic acid grafted- reduced graphene oxide (PAA-rGO), sheer storage modulus (SSM), cellulose nanocrystals (CNC), Phrenyl-naphthalene-diimide (Ph-NDI), pyrene-functionalized polyamide, a polyimide and pyrene-functionalized gold nanoparticles (Py-PDI-P-AuNPs), polystyrene (PS), poly(styrene-co-4-cyanostyrene) (PSCN) and poly(styrene-co-2-vinylnaphthalene) (PSNP), Surface profilometry (SP), Methylimidazole/methylthioether, (MI/MT-SiO1.5), silsesquioxane (SQ).
